# The risk of vector transmission of *Trypanosoma cruzi* remains high in the State of Paraná

**DOI:** 10.1590/0074-02760230226

**Published:** 2024-06-10

**Authors:** João Vitor S Trovo, Michele Martha Weber-Lima, Bianca Prado-Costa, Giullia F Iunklaus, Andrey J Andrade, Thadeu Sobral-Souza, Renata L Muylaert, Larissa M Alvarenga, Max Jean O Toledo

**Affiliations:** 1 Universidade Estadual de Maringá, Centro de Ciências da Saúde, Programa de Pós-Graduação em Ciências da Saúde, Maringá, PR, Brasil; 2 Universidade Federal do Paraná, Departamento de Patologia Básica, Programa de Pós-Graduação em Microbiologia, Parasitologia e Patologia, Curitiba, PR, Brasil; 3 Universidade Federal do Mato Grosso, Instituto de Biociências, Departamento de Botânica e Ecologia, Laboratório de Macroecologia, Cuiabá, MT, Brasil; 4 Massey University, Hopkirk Research Institute, Molecular Epidemiology and Public Health Laboratory, Palmerston North, New Zealand.; 5 Secretaria Estadual da Saúde, Curitiba, PR, Brasil

**Keywords:** Triatominae, ecological niche models, climate, landscape, Chagas disease

## Abstract

**BACKGROUND:**

Monitoring and analysing the infection rates of the vector of *Trypanosoma cruzi*, that causes Chagas disease, helps assess the risk of transmission.

**OBJECTIVES:**

A study was carried out on triatomine in the State of Paraná, Brazil, between 2012 and 2021 and a comparison was made with a previous study. This was done to assess the risk of disease transmission.

**METHODS:**

Ecological niche models based on climate and landscape variables were developed to predict habitat suitability for the vectors as a proxy for risk of occurrence.

**FINDINGS:**

A total of 1,750 specimens of triatomines were recorded, of which six species were identified. The overall infection rate was 22.7%. The areas with the highest risk transmission of *T. cruzi* are consistent with previous predictions in municipalities. New data shows that climate models are more accurate than landscape models. This is likely because climate suitability was higher in the previous period.

**MAIN CONCLUSION:**

Regardless of uneven sampling and potential biases, risk remains high due to the wide presence of infected vectors and high environmental suitability for vector species throughout the state and, therefore, improvements in public policies aimed at wide dissemination of knowledge about the disease are recommended to ensure the State remains free of Chagas disease.

Chagas disease (CD) or American trypanosomiasis has the haemoflagellate protozoan *Trypanosoma cruzi* (Chagas, 1909) as its etiological agent and affects about 6-7 million people worldwide, mainly in the Americas, it is most commonly transmitted by Triatominae (Hemiptera, Reduviidae) insect vector, when humans come into contact with the vector’s contaminated faeces, carried out after a blood meal. There are also other routes of transmission, such as blood transfusion, organ transplantation, and congenital transmission.^1^ ^,^ ^2^ ^,^ ^3^ According to the National System of Notifiable Diseases (Sistema de Informação de Agravos de Notificação - SINAN), in Brazil alone, from 2012 to 2020, there were 2,458 cases, 533 between 2020 and 2021. The cases cluster mainly in the North Region of Brazil, and a total of 1,746 deaths due to the disease were reported.^4^ Currently, oral transmission is the main route of human infection by *T. cruzi*, through the consumption of food contaminated by the pathogen, followed by vector transmission that occurs through human contact with the feces of the infected vector.^5^

Chagas disease vectors are distributed in five tribes and 18 genera, covering about 157 species (154 living species and three fossils),^6^ just 12 present epidemiologic importance.^7^ ^,^ ^8^ In Brazil there are about 68 known species of triatomines to date.^7^ Some are epidemiologically important due to their behavioural characteristics, especially *Triatoma infestans* Klug, 1834. As *T. infestans* is an introduced species in Brazil and has adapted to intradomicile, its control was mostly made possible through intense entomological surveillance and chemical control actions.^8^ ^,^ ^9^ ^,^ ^10^ However, the ecological niches vacated by *T. infestans* are being occupied by other native species with the potential for domiciliation, such as: *Triatoma brasiliensis* Neiva, 1911, *Triatoma pseudomaculata* Corrêa & Espínola, 1964, *Panstronsgylus megistus* Burmeister, 1835, *Triatoma maculata* Erichson, 1848, *Triatoma rubrovaria* Blanchard, 1843, and *Triatoma sordida* Stål, 1859.^7^ ^,^ ^11^ ^-^ ^18^ *Panstrongylus megistus* has become a particularly relevant vector because it has a great ability to adapt to artificial ecotopes and to intradomicile in rural areas and it can even be found in urban areas.^19^ ^,^ ^20^ *T. sordida* and *Rhodnius neglectus* Lent, 1954, are also of great epidemiological importance as they are found in and around rural homes, with high rates of *T. cruzi* infection, up to 43%.^21^ ^,^ ^22^ In the State of Paraná, southern Brazil, in addition to the four species (*T. infestans*, *P. megistus*, *T. sordida*, and *R. neglectus*) mentioned above, there are records of the occurrence of six additional species: *Panstrongylus geniculatus* Latreille, 1811, *Rhodnius prolixus* Stål, 1859, *Rhodnius domesticus* Neiva & Pinto, 1923, *P. tibiamaculatus* Pinto, 1926, *Cavernicola pilosa* Barber, 1937, *Microtriatoma borbai* Lent & Wygodzinsky, 1979.^11^ ^,^ ^23^ ^,^ ^24^

Captures of these triatomine insects may occur both by passive and active surveillance. Passive surveillance occurs when the resident finds the triatomine in their home and sends it to the entomological surveillance of the municipality for notification. On the other hand, active surveillance occurs when the health agents themselves carry out an active search in the residence, notifying the occurrence of triatomines and capturing specimens. The captured insects are sent to the State Department of Health (“Secretaria Estadual da Saúde” - SESA) for identification at the species level, life stage (adult and nymph) and analysis of the presence of *T. cruzi* infection.^12^ ^,^ ^25^

Ecological niche modelling (ENM) is a methodology that uses mathematical algorithms to relate occurrence data with environmental variables to infer potential areas of species occurrence. This approach has been used for several diseases such as hantavirus and visceral leishmaniasis,^26^ ^,^ ^27^ and also for CD in different regions, as it is meaningful for understanding the environmental requirements and geographic distribution of vectors leading to a better understanding of the epidemiological aspects of the disease.^28^ Costa et al. used the ENM methodology for the first time to analyse Brazilian species of triatomines.^29^ ENM predictions for Brazil indicate that the distribution of *R. neglectus* expanded towards the west and northwest of the Brazilian Cerrado.^30^ In the State of Paraná, in a study carried out by Ferro e Silva et al.,^23^ models for the years 2007-2013 predicted that the municipalities in the northwest, north and northeast regions of the state have higher values for climatic and landscape potential habitat suitability for the occurrence of triatomines (hereafter risk of *T. cruzi* vector occurrence). ENM studies can also be necessary for analysing models that estimate the effects of climate change on species distribution.^31^

The present study aimed to investigate the risk areas for CD in the State of Paraná and evaluate the risk of vector transmission of *T. cruzi* based on a decade of triatomine occurrence data (2012 to 2021) and discuss the main patterns in temporal variation in vector distribution and infection rates within the state. Based on previous research and surveillance, it was expected that potential habitat suitability for vectors would be consistently higher in the northwest for the current period, peaking at north and northeast regions of the state, varying according to landscape and climate predictors. Another objective was to estimate and discuss potential sampling biases in the occurrence data that matched geospatial coordinates of municipalities. The implications of model predictions and potential biases for estimating the risk of vector transmission of the CD agent to humans based on surveillance data at the municipality level are discussed.

## MATERIALS AND METHODS

**Fig. 1:**
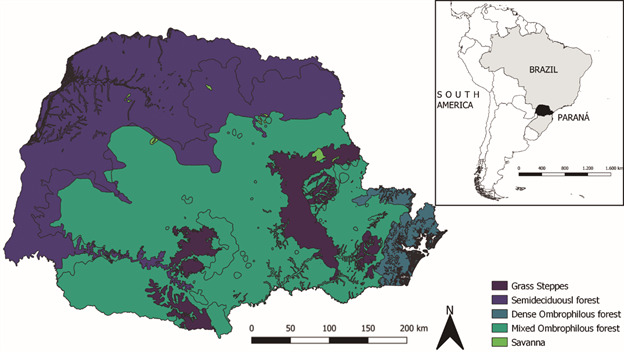
geographical location, main vegetation types and forest remnants of the Atlantic Forest biome of the State of Paraná, southern Brazil. Software: QGIS. Source: IBGE^32^.

*Study area* - The State of Paraná has a population of about 11.6 million inhabitants spread over approximately 200,000 km², thus having a population density of 57.4 inhabitants per square kilometre.^32^ It is located within the Atlantic Forest (covering 97.8% of the State) and non-forest formations. Within the Atlantic Forest biome, five major phytogeographic units stand out: Dense Ombrophilous Forest, Mixed Ombrophilous Forest, Semideciduous, Steppes (or Fields) and Cerrado (or Savannah) (Fig. 1).^33^

The Dense Ombrophilous Forest has a large and complex collection of biological forms and rainfall distributed throughout the year, influenced by the warm and humid air masses of the Atlantic Ocean. The Araucaria Mixed Rain Forest, which has a certain complexity of species, but with a predominance of araucaria (*Araucaria angustifolia*), well-distributed rainfall throughout the year and vegetation influenced by regular frosts in winter. The Semideciduous Forest (or seasonal) has low precipitation and occasional frosts, forcing its vegetation to lose its leaves. The Steppes (Campos) have, for the most part, grassy vegetation and gallery forests (close to riverbanks). And finally, Savannah or Cerrado, characteristic of the semi-arid climate of Brazil.^34^ Paraná’s climate can be subdivided into three subtypes: temperate humid with hot summers, sub-humid with little water deficiency, megathermal and humid subtropical with dry winter.^35^

*Specimens collection and construction of georeferenced maps* - The triatomines were obtained from collections carried out in the routine of the SESA, being collected and forwarded by the population to the “Secretaria Municipal de Saúde” (SMS) and from these to the “Regional de Saúde” (RS), where there is a screening of the specimens, which are forwarded to the “Núcleo de Vigilância Entomológica” (NVE) and/or to the “Divisão de Doenças Transmitidas por Vetores” (DVDTV), to be identified and analysed for presence of *T. cruzi*.

The taxonomic identification of triatomines was carried out according to the dichotomous key proposed by Lent & Wygodzinsky^7^ 1979 and Jurberg et al.^36^

The analysis of the infection rate by *T. cruzi* was carried out using the conventional method, direct fresh examination of the intestinal contents, through abdominal compression after the insect was anesthetised in the presence of cotton wool soaked in chloroform or ether. Excreta (faeces and urine) are deposited on a slide with 0.9% NaCl physiological solution and an overlapping cover slip (20 x 20 mm). The microscopic fields were observed at 400X magnification using an Olympus microscope.

Specimens were notified according to year of notification, municipality, place of capture (intradomicile and peridomicile), developmental stage (nymph or adult), sex and positivity for trypanosomatids. This information was tabulated in Excel and georeferenced using the data analysis program using QGIS version 3.22.1. In order to create occurrence maps, the municipality’s centroid was used to assign a geospatial location (WGS 84) for the triatomine capture data using the Realcentroid plugin (1.0.3).

Once in possession of this information, the point of capture of the specimens was plotted on the map of the State of Paraná with the municipal grid, made available by the “Instituto Brasileiro de Geografia e Estatística” (IBGE),^32^ thus obtaining maps of the capture sites for each species in the state.

*Ecological niche models* - For predicting environmental suitability, the modelling workflow from Ferro e Silva et al.^23^ was applied. The covariate data used in model building was composed by a selection from 19 climate variables and five landscape variables. A factorial analysis similar to the methodology of Sobral-Souza et al.^37^ was carried out to obtain the variables that were less correlated, but that explained a greater environmental variation in the study area. After this step, the following climate variables were selected: isothermality, annual temperature variation, temperature in the hottest quarter, precipitation in the wettest quarter and precipitation in the coldest quarter. All climatic variables were extracted from the WorldClim dataset v 1.4 (https://www.worldclim.org).

From the five landscape variables extracted from Earthenv (http://www.earthenv.org/texture), those that were most related to the effects of landscape fragmentation on the dispersal of triatomines, due to their association with human occupations, were selected. Other variables, such as the type of vegetation cover, species and state of vegetation preservation are also likely to influence the areas where triatomines occur. Therefore, vegetation cover and functional connectivity were selected for landscape model building.

For a better observation and comparison of data and predicted suitability values considering municipality centroids, 1000 random points were generated throughout the state. The Rstudio (2021.09.0) and R (R Core Team)^38^ were used in analyses. Distributions of observations in municipalities were compared with random points using violin plots, which combine the boxplot graph with density data. In addition to demonstrating in which indices each captured species was most found and also the capacity of the previously generated models to divide the species in relation to the indices where they were more frequent, expectedly higher suitability values.

*Statistical analysis* - Because of limitations of data collection and attribution of geospatial locations of triatomines to municipality centroids, the data was evaluated for potential sampling biases. The SAMPBIAS package^39^ was used, which uses a Bayesian approximation to calculate the accessibility bias of a species occurrence database. SAMPBIAS runs in the R language. As input data, information on species (occurrence), year and municipality centroid location (latitude and longitude) were entered for each of the 1,750 occurrences of triatomines. SAMPBIAS estimates four sampling bias drivers based on Bayesian inference, namely highways, cities, rivers, and airports.

## RESULTS

**Fig. 2:**
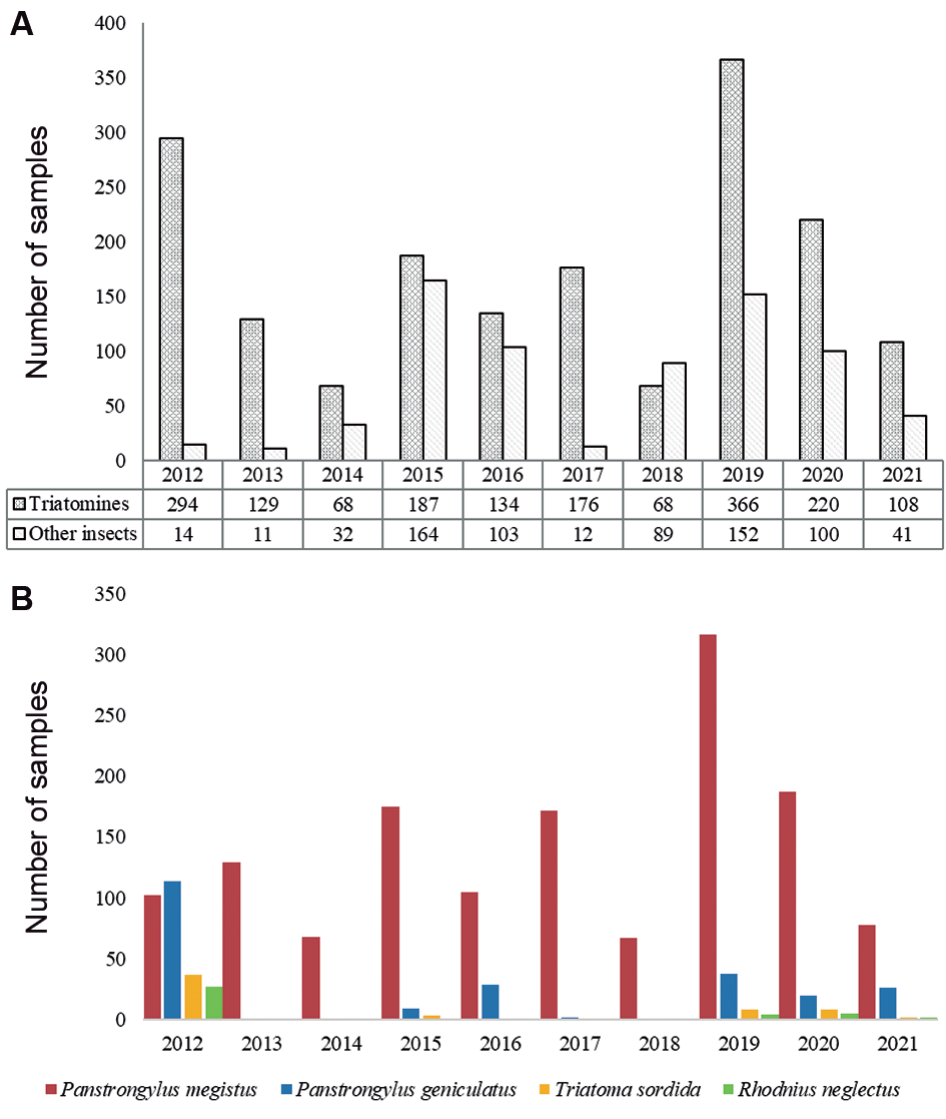
occurrence of triatomines sampled during entomological surveillance from 2012 to 2021 in the State of Paraná, southern Brazil. (A) Insect notifications per year by passive and active surveillance. (B) Notifications by species of triatomines *Panstrongylus megistus*, *Panstrongylus geniculatus*, *Triatoma sordida* and *Rhodnius neglectus* from 2012 to 2021. Source: Divisão de Doenças Transmitidas por Vetores/Secretaria Estadual da Saúde/Paraná.

In the period from 2012 to 2021, totalling 10 years, 1,750 specimens of triatomines were recorded in the State of Paraná. In 2019, the highest number of notifications (n = 366) was recorded, an increase mostly related to the high number of samples from a colony in Nova Aurora municipality (n = 123) (Fig. 2A).

In Paraná, six species from entomological surveillance were recorded, namely: 80% of *P. megistus* (n = 1,399), 14% of *P. geniculatus* (n = 238), 3% of *T. sordida* (n = 58), and 2% of *R. neglectus* (n = 39). Less than 1% (n = 14) of the samples lacked species-level classification, all belonging to the genus *Rhodnius* (classified within the cis-*prolixus* group). These specimens were disregarded in species-level analyses. Two specimens of *P. tibiamaculatus*, formerly *T. tibiamaculata*,^40^ were also recorded in the period but not included in this analysis. Other insect species that are confused with triatomines were also recorded during surveillance (Fig. 2A).

*Panstrongylus megistus* was the only species found in all ten years of the study period, and also the most frequently captured, except in 2012 when *P. geniculatus* comprised most captures. The year 2012 was also the only one in which all four most prevalent species were found. The species referred to as *R.* cis-*prolixus* group was recorded in 2012. In 2013, 2014 and 2018, only the species *P. megistus* was recorded (Fig. 2B). The two specimens of *P. tibiamaculatus* were recorded in 2016 and 2017.

**Fig. 3:**
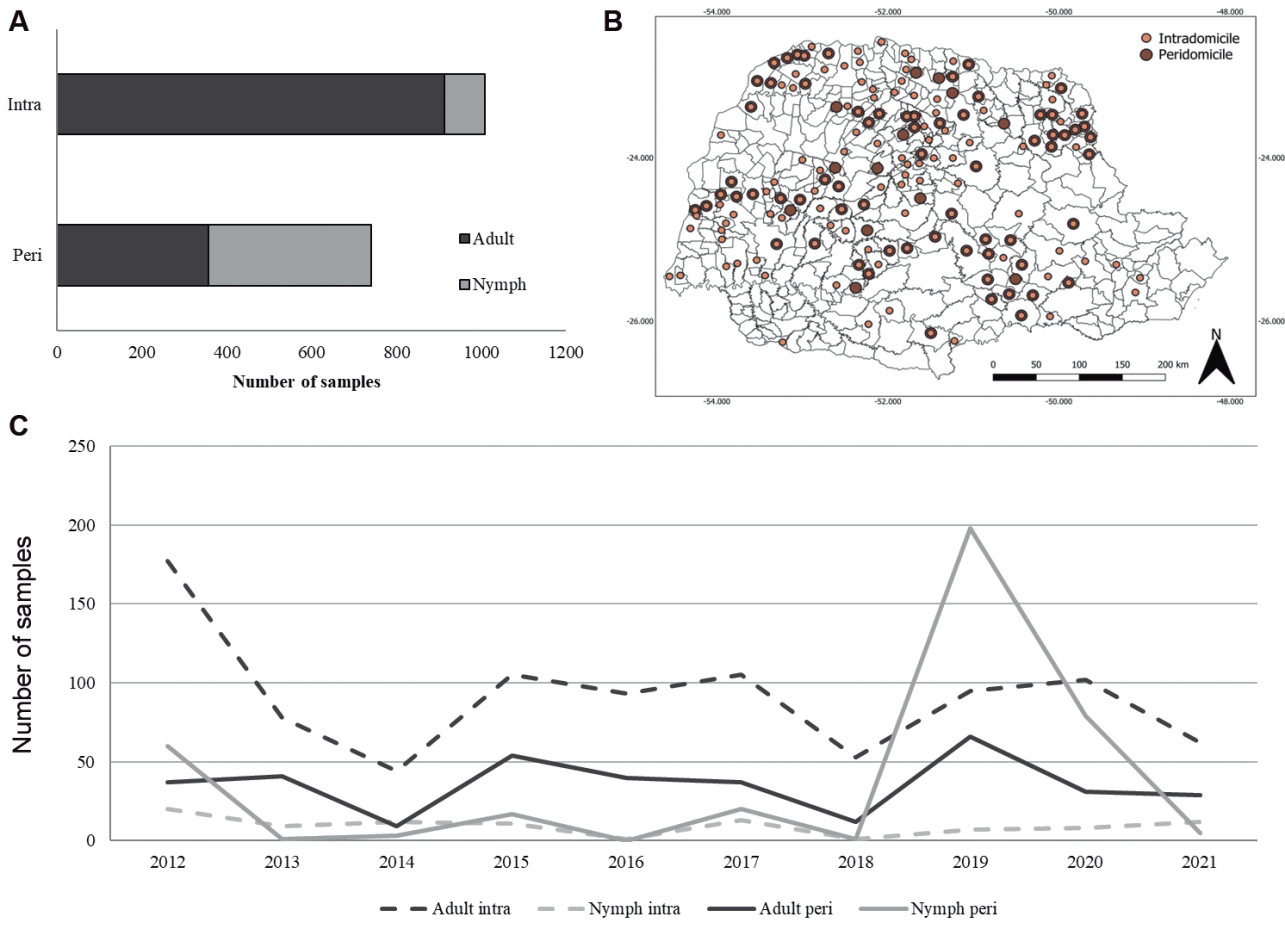
relation of adult or nymph insects reported in the intra and peridomicile from 2012 to 2021 in the State of Paraná, southern Brazil. (A) Comparison rate of adults and nymphs collected in the intra and peridomicile. (B) Dispersion of the triatomines collected in the intra and peridomicile by municipality. (C) Analysis of relation adults and nymphs in the intra and peridomicile. Source: Divisão de Doenças Transmitidas por Vetores/Secretaria Estadual da Saúde/Paraná.

Of the total number of notified samples, 57.7% (n = 1,010) were found in the intradomicile and 42.3% (n = 740) in the peridomicile, also 72.7% (n = 1,272) were adults while 27.4% (n = 478) were nymphs (Fig. 3A). Regardless of the place of capture, whether intradomicile, peridomicile, or both, the insects were distributed throughout the State of Paraná (Fig. 3B). The highest number of occurrences was of adult insects, followed by nymphs, both in the intradomicile during most of the study period. In 2019, however, nymphs in the peridomicile comprised most of the occurrences (Fig. 3C).

**Fig. 4:**
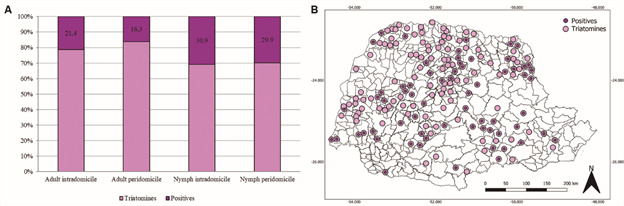
positivity of triatomines for *Trypanosoma cruzi* in the State of Paraná, south Brazil from 2012 to 2021. (A) Infection rates of synanthropic triatomines captured in the intra and peridomicile according to the developmental stage (adult or nymph). (B) Points of occurrence of negative and positive insects for *T. cruzi*. Source: Divisão de Doenças Transmitidas por Vetores/Secretaria Estadual da Saúde/Paraná.

The overall infection rate for *T. cruzi* was 22.7% (n = 398/1,750). In the peridomicile, 23.3% (n = 173/740) of the insects were positive for *T. cruzi* and in the intradomicile, 22.3% (n = 225/1,010). Moreover, the infection rate for *T. cruzi* was higher in nymphs than in adults. The nymphs showed a similar percentage of positivity in the intra 30.9% (n = 29/94) and in the peridomicile 29.9% (n = 115/384). Adults were more often positive in the intradomicile 21.4% (n= 196/916) when compared with the peridomicile 16.3% (n = 58/356) (Fig. 4A). Infected triatomines were distributed throughout most of the State of Paraná (Fig. 4B).

**Fig. 5:**
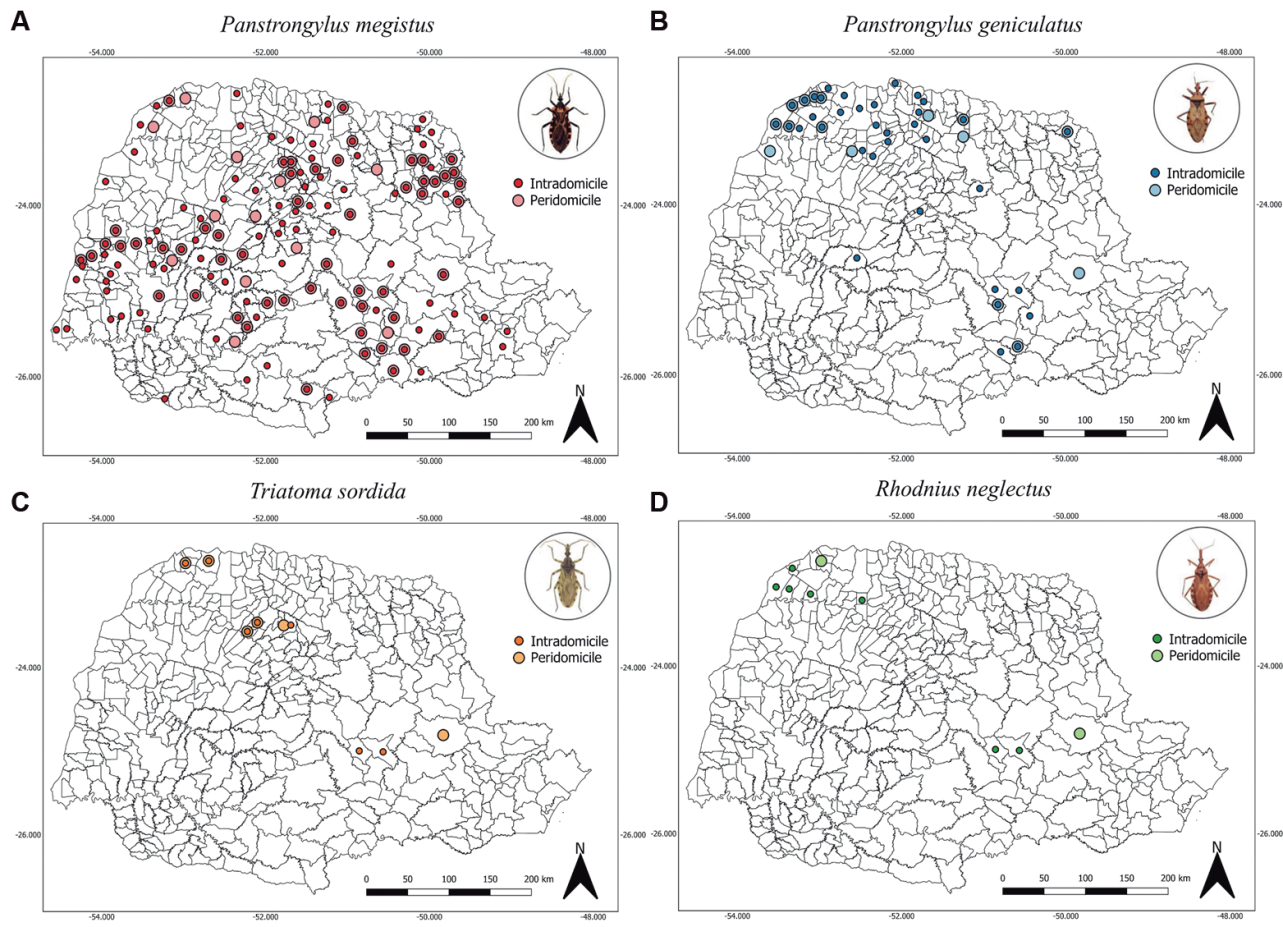
occurrence points in intra and peridomicile of triatomines notified by species in the State of Paraná, southern Brazil, from 2012 to 2021. (A) *Panstrongylus megistus*. (B) *Panstrongylus geniculatus*. (C) *Triatoma sordida*. (D) *Rhodnius neglectus*. Source: Divisão de Doenças Transmitidas por Vetores/Secretaria Estadual da Saúde/Paraná.

Observing the species occurrence maps, *P. megistus* presented a wide dispersion throughout the State of Paraná, whereas the species *P. geniculatus* presented a distribution more restricted to the northern region of the state. Although rarer, *T. sordida* and *R. neglectus* were found in the northwest and central-east regions (Fig. 5).

**Fig. 6:**
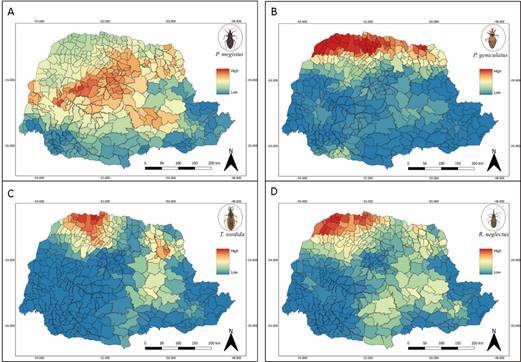
suitability for the occurrence of triatomines within municipalities of the State of Paraná predicted using climate. (A) *Panstrongylus megistus*. (B) *Panstrongylus geniculatus*. (C) *Triatoma sordida*. (D) *Rhodnius neglectus*. Source: Divisão de Doenças Transmitidas por Vetores/Secretaria Estadual da Saúde/Paraná.

Considering the four most prevalent species and the climate layer, the areas of highest climatic suitability for *P. megistus* differed from the areas of climatic suitability for *P. geniculatus*, with the first species showing broad suitability for several regions of Paraná (Fig. 6A) and the second with suitability more concentrated in the northern region of the state (Fig. 6B). Differing, in turn, from the potential distribution of habitat predicted for *T. sordida* and *R. neglectus*. These last two species presented similar climatic suitability, with higher values for the northwest region and several areas in the central-east of the state with intermediate suitability (Fig. 6C-D).

**Fig. 7:**
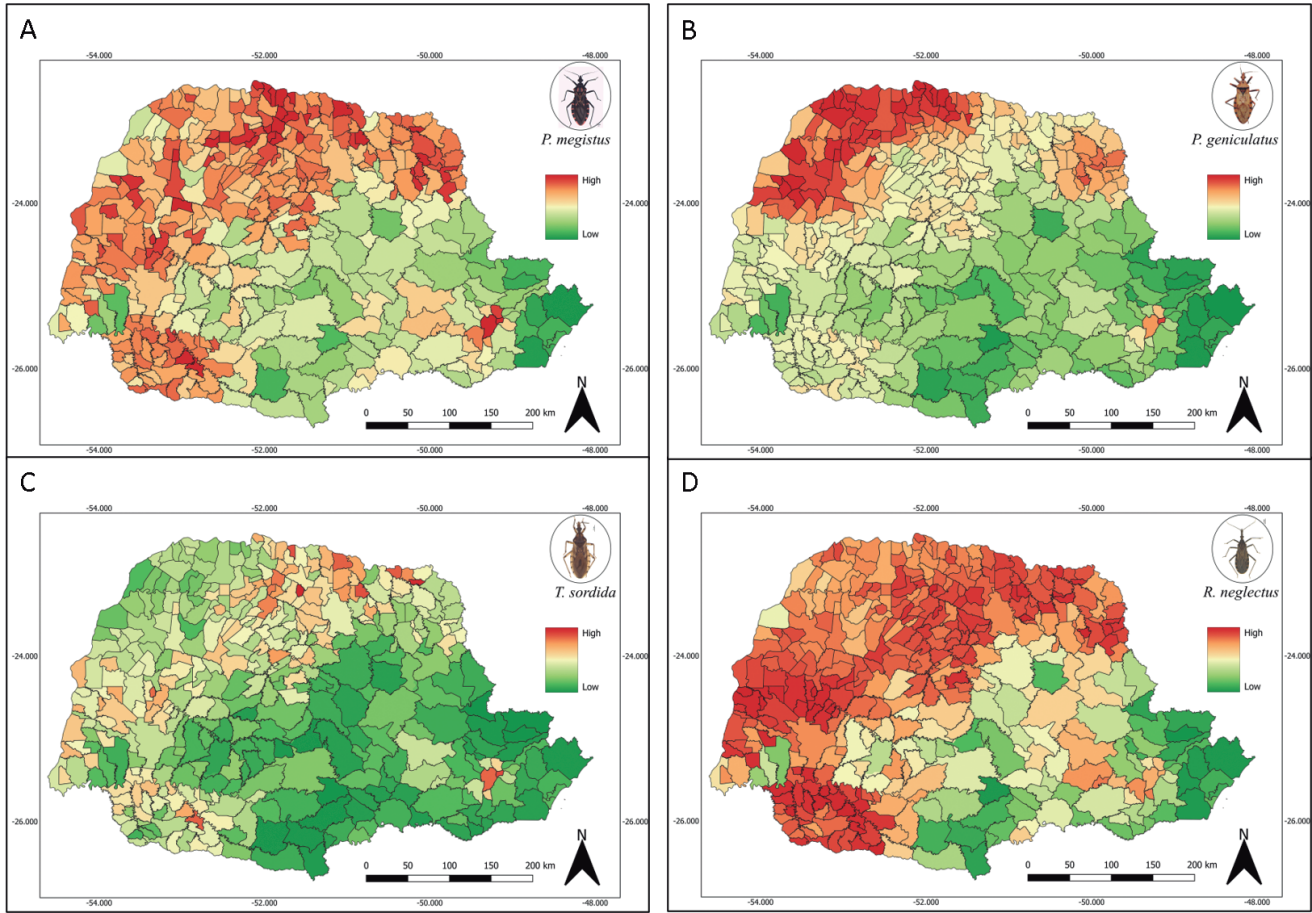
suitability for the occurrence of triatomines within municipalities of the State of Parana predicted using landscape covariates. (A) *Panstrongylus megistus*. (B) *Panstrongylus geniculatus*. (C) *Triatoma sordida*. (D) *Rhodnius neglectus*. Source: Divisão de Doenças Transmitidas por Vetores/Secretaria Estadual da Saúde/Paraná.

Thinking about potential distribution taking into account the landscape ENMs, *P. megistus*, *P. geniculatus* and *R. neglectus* showed wide distribution throughout the state (Fig. 7A, B and D). On the other hand, *T. sordida* seems to be less adapted to the southeastern region of Paraná (Fig. 7C).

**Fig. 8:**
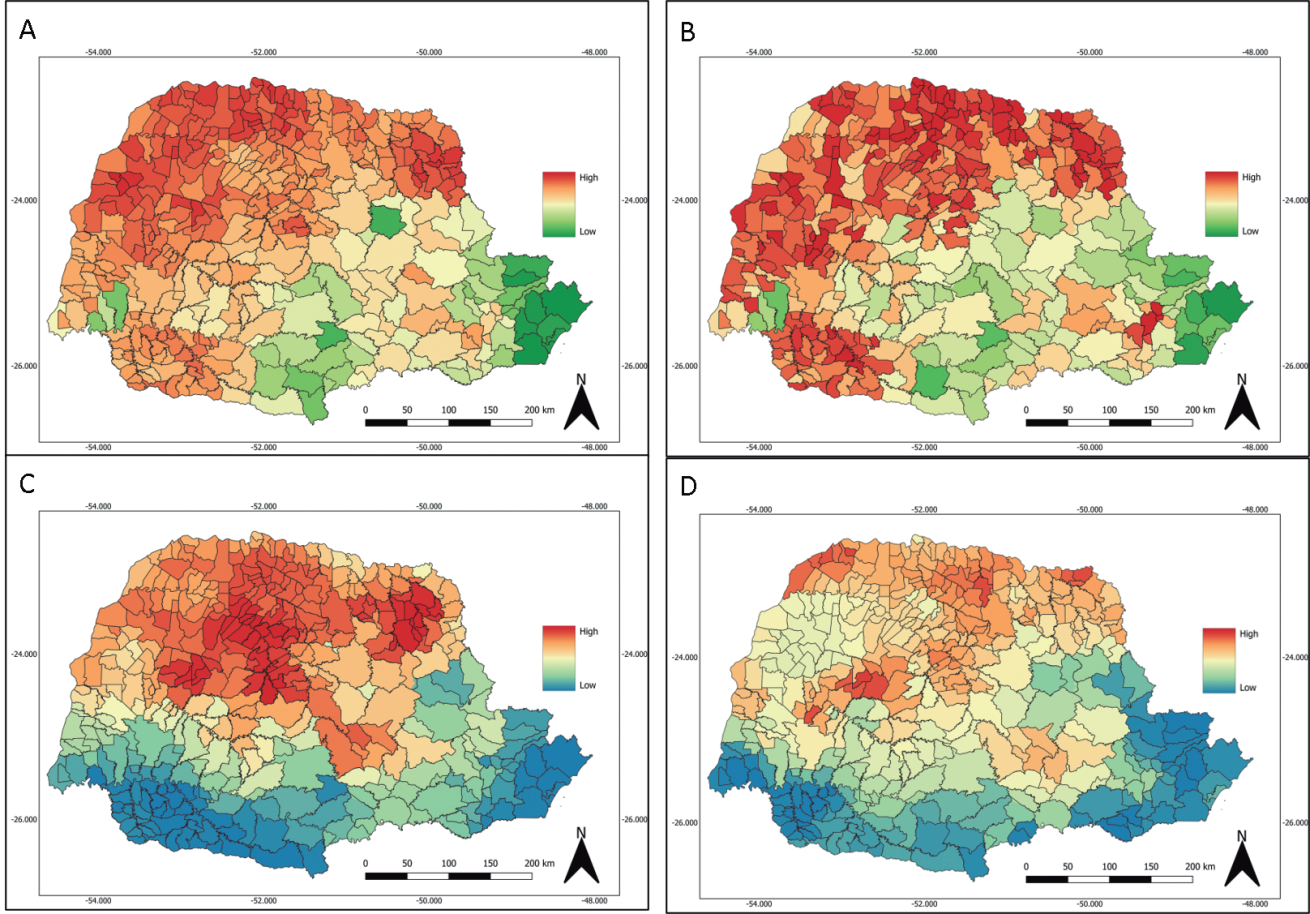
average suitability values for the occurrence of triatomines within municipalities of the State of Paraná predicted using climatic and landscape environmental layers. (A) Landscape-only models (date: 2007 to 2013); (B) Landscape-only models (date: 2012 to 2021); (C) Climate-only models (date: 2007 to 2013); and (D) Climate-only models (date: 2012 to 2021). Source: Ferro e Silva et al*.* ^23^ ^)^ for Figs A and C, and Divisão de Doenças Transmitidas por Vetores/Secretaria Estadual da Saúde/Paraná for Figs B and D.

Regardless of the species, the regions with the highest occurrences correlate with the areas of greatest environmental suitability for these insects, which are represented on the maps by warmer colours, both for the climate ENMs (Fig. 8B) and for the landscape ENMs (Fig. 8D). In comparison with the previous study by Ferro e Silva et al.^23^ when overlapping the climatic and landscape layers for the different species found, the current results show very similar patterns for both climate and landscape, with a slight reduction in the maximum values for potential habitat suitability for climate and an increase in maximum values for landscape suitability (Fig. 8).

Among the 10 municipalities with the highest number of captured triatomines, three showed high suitability for both climate and landscape covariates, five showed high and medium suitability, and one medium and high suitability, respectively, for climate and landscape. Only one municipality showed average suitability for both climate and landscape. None of the ten municipalities with the highest occurrences showed low environmental suitability (Table).

**TABLE:** Environmental suitability in the ten municipalities with the highest number of occurrences of triatomines in the State of Paraná, southern Brazil, from 2012 to 2021

Municipality	Captured triatomines	Climate SI	Climate suitability	Landscape SI	Landscape suitability
Guamiranga	135	0.79	high	0.57	medium
Nova Aurora	131	0.72	high	0.79	high
Boa Esperança	106	0.99	high	0.83	high
Prudentópolis	97	0.82	high	0.35	medium
Ivaí	57	0.87	high	0.54	medium
Nova Londrina	56	0.80	high	0.83	high
Roncador	52	0.75	high	0.62	medium
São Pedro do Paraná	51	0.72	medium	0.88	high
Rebouças	49	0.29	medium	0.53	medium
Apucarana	45	0.86	high	0.71	medium

SI: suitability index values [> 0.74 (high); from 0.26 to 0.73 (medium); < 0.25 (low) ].

**Fig. 9:**
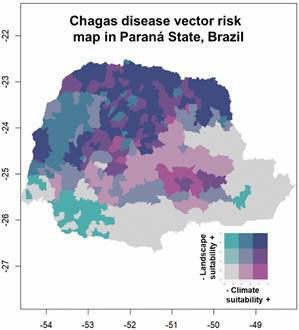
risk map for the occurrence of triatomines within municipalities of the State of Paraná predicted based on climate and landscape ecologic niche models (ENM) and zonal statistics for municipalities, from 2012 to 2021.

The north, northeast, and northwest of the State of Paraná are more suitable for the occurrence of the invertebrate hosts (kissing bugs) of *T. cruzi*. This is demonstrated by the high suitability, both for climatic and landscape factors, of municipalities marked in dark blue. Other municipalities in the northwest region have high climate suitability, but low landscape suitability, thus demonstrating that they are municipalities with a lower risk of transmission. The municipalities in the south and southeast of the state present a low risk for the presence of triatomines, therefore being the lowest risk of transmission [Fig. 9, and Supplementary data (Table)].

**Fig. 10:**
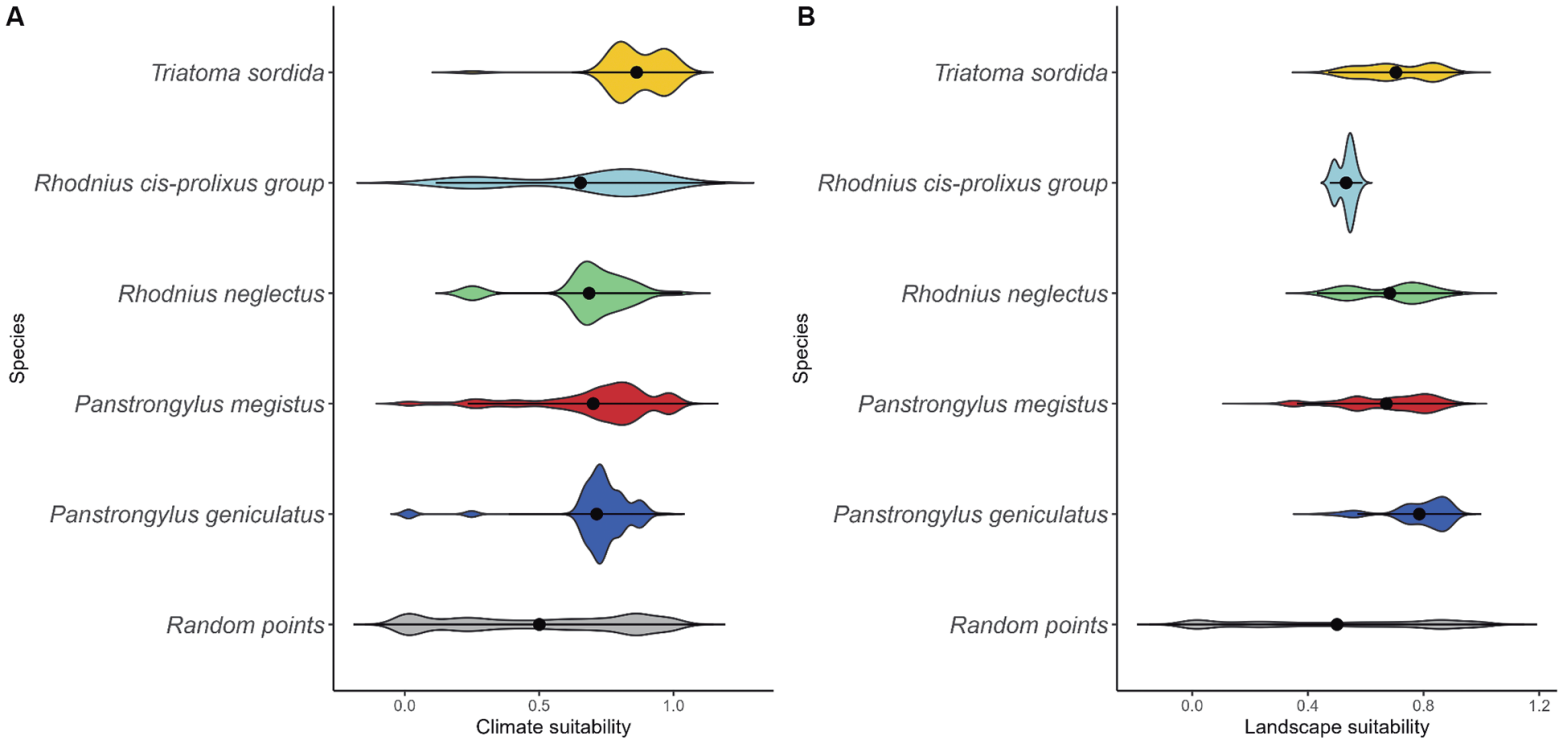
distribution of triatomine species according to climate (A) and landscape (B) suitability indices. The black dots in the graphs represent the average suitability values, and violin distribution represents the density of suitability values, varying between zero and one. State of Paraná from 2012 to 2021.

Average potential habitat suitability (ADE) values remain higher than the average expected for random locations for all vector species for both landscape and climate models (Fig. 10). The mean ADE values for climate ranged from 0.70 to 0.84, with the species that had the highest climate mean being *T. sordida* (0.84), followed by *P. megistus* (0.76), *P. geniculatus* (0.72) and finally *R. neglectus* (0.70). As for the landscape ADE values, the averages ranged from 0.54 to 0.83, with *P. geniculatus* presenting the highest average (0.83), followed by *R. neglectus* (0.74), *P. megistus* (0.71) and lastly, *T. sordida* (0.68).

**Fig. 11:**
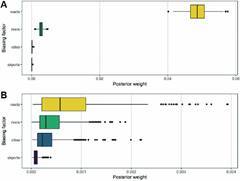
influence of sampling bias in the triatomine occurrence database. Accessibility bias estimates regarding the sampling of triatomines in the State of Paraná from 2012 to 2021. Sample analysis (A). Random analysis (B). Software: RStudio (2021.09.0)/ SAMPBIAS.

The sampling bias analysis estimated a positive effect of highways on the sampling of triatomines, with a weight bias of 0.04. However, for the other analysed biases (rivers, cities, and airports) weights lower than 0.002 were obtained (Fig. 11A). A more similar distribution for the biases was observed with a random distribution, with weights of 0.0007 for highways and 0.00009 for airports (Fig. 11B). The triatomine occurrence data in the State of Paraná are biased towards the most accessible locations (close to highways) (Fig. 11A) since the posterior weight of this variable is considerably higher than what would be expected for a random spatial sampling [Fig. 11B, and Supplementary data (Table)].

## DISCUSSION

In the 10-year period of the current study, a total of 1,750 triatomines were found, equivalent to 61.7% of the number of specimens found in the previous period analysed by Ferro e Silva et al.^23^ which recorded 2,662 occurrences, even though in a shorter study period of 7 years (2007-2012) [Supplementary data (Table)] demonstrating a reduction in notifications made in more recent years. However, the exact most prevalent species were recorded, with *P. megistus* being the most commonly found, followed by *P. geniculatus*, *T. sordida* and *R. neglectus*. Falavigna-Guilherme et al.^41^ in a study carried out between 1996 and 2000, observed that the species most often found in Paraná, in decreasing order of prevalence, were *T. sordida*, *P. megistus* and *R. neglectus*. It should be noted that this study was based on the active surveillance of triatomines.

This study reports the appearance of *R. prolixus*, also previously reported by Ferro e Silva et al.^23^ Although *R. prolixus* is native to the Andes and is one of the main vectors of CD in Venezuela and Colombia, there are records of this species in the Amazon Forest; however, it can also be target of erroneous identification, due to the significant morphological similarity among the species of the genus *Rhodnius*.^42^ Hernández et al.^43^ report misidentifications related to *R. prolixus* with *R. neglectus*, *R. robustus* and other species of the genus *Rhodnius*, classifying them in a group called cis*-prolixus* of great epidemiological and distributional importance. There are limitations in identifying the morphological variability among the different species of the genus *Rhodnius*, resulting in inconsistent identification. Because of that, molecular markers are being more commonly used to differentiate such species considered challenging to identify solely through morphological and isoenzymatic characteristics.^43^

Unlike previous studies carried out in Paraná,^21^ ^,^ ^23^ ^,^ ^41^ recently *P. tibiamaculatus* was recorded on the coast and in the interior of the state. This species presents a geographic distribution more restricted to the Atlantic Forest and is frequently attracted by light but rarely colonises houses.^13^ However, it was found in peridomestic areas where contamination of the sugar cane juice was detected, causing several cases of oral transmission recorded in Santa Catarina,^44^ a state that share borders with Paraná. This species has epidemiological importance as it has already been found infesting domiciliar areas, and the frequent invasion of homes by infected triatomines of this species, from forests close to houses, as observed in the municipality of Salvador (Bahia), indicates a potential risk of transmission of *T. cruzi* to the inhabitants of the area.^45^ In addition to Bahia and Santa Catarina, this species is present in eight other Brazilian states, including Paraná.^11^ The return of its appearance in Paraná reinforces that continuous epidemiological surveillance must be carried out in areas where domestic transmission is controlled, but enzootic transmission persists.

Regarding the place where these insects were found, the species remained the most frequently found in the intradomicile 57.7% (n = 1,010/1,750) which can be explained by the passive surveillance carried out in the state, where the population collects samples. This has also been observed by other authors.^23^ ^,^ ^46^ The presence of different triatomines in the peri and intradomicile can be an indication of the potential for domiciliation of vectors related to the species, with some being more adapted to wild settings and others more adapted to domiciles.^47^ ^,^ ^48^

The vast majority of specimens, 72.7% (n = 1,272/1,750), were adults, probably due to the greater dispersal capacity of this developmental stage, as they are winged and have larger dimensions compared to nymphs, which are wingless and smaller, in addition, to dispersing in search of food.^42^ Adults may come from forest areas, attracted by the light, making it easier for residents to find them^48^ ^,^ ^49^ and then they can be found in isolation, without having yet formed colonies, which is why an active search is recommended to verify a possible domiciliation.^25^ The developmental stage of specimens related to triatomine surveillance is very important because when nymphs are found, the colony is close. Because they do not fly, nymphs have a much lower displacement potential than the adult form.^48^

In the present study, the overall rate of *T. cruzi* infection was 22.7% (n = 397/1,750), slightly higher than the rate in the previous study of 19.7% (n = 486/2,662), which signals an important increase in the positivity rate, especially considering that a smaller number of samples was examined.^25^ The triatomine positivity ratio is an important criterion for triggering the surveillance of human cases and it is recommended that parasitological examinations be carried out in all residents where positive triatomines are found.^25^ It should be noted that not all triatomines presented as negative on the map (Fig. 4) are insects without the presence of the parasite, as some samples are degraded and not suitable for parasitological analysis, often arriving dry to the lab. Thus, it can be estimated that positivity is likely higher than what was detected. It would be desirable to assess the correlation between *T. cruzi* infection and triatomine species, but this was beyond the scope of the present study and would require an analysis design that corrects for the uneven sampling of specimens and the overwhelming majority of *P. megistus*.

*Panstrongylus megistus* showed a wide dispersion throughout the State of Paraná. According to the Ministry of Health, this species has potential for domiciliation.^12^

Over the 10 years of the current study, this species prevailed over the others, corresponding to 80.0% (n = 1.399/1.750) of all specimens captured, except in 2012 when it was surpassed by *P. geniculatus*, and presented an increasing number of specimens and oscillating in the period. Unlike the seven-year period of the previous study, in which the species showed a decreasing number of captures. The increase in the capture of this species in recent years, and the return of species diversity in the last three years of the current study requires attention from health agencies, mainly due to the epidemiological importance of *P. megistus* in the transmission of *T. cruzi*.^42^

For *P. megistus*, more adult specimens were also found when compared to nymphs. However, 29.1% (n = 407/1,399) of the total specimens of this species were nymphs and 80% (326/407) were colonising the peridomicile. This species had the highest infection rate for *T. cruzi* 27.0% (n = 378/1,399) among the species captured, similar to the previous study whose rate was 24.7%.^23^ The data demonstrate that *P. megistus*, despite being considered wild in southern Brazil, has been showing domestic characteristics for some time.^50^ ^,^ ^51^ ^,^ ^52^ ^,^ ^53^ In Paraná, according to the data presented, several colonies were found with a high number of nymphs demonstrating this domiciliation in the Southern region of Brazil. Since this species has greater vectorial capacity and greater ability to form intradomiciliary colonies, a higher proportion of specimens of this species infected with *T. cruzi* increases the risk of transmission of the protozoan.^42^ ^,^ ^54^

The species *P. geniculatus* presented a distribution more restricted to the northern region of the state. Although it is a species with wild behaviour, adults can be attracted to light and are often found inside houses.^48^ *P. geniculatus*, was the second species with the highest occurrence, corresponding to 14% (n = 238/1,750) of the captured specimens and more adults (n = 186/238) than nymphs (n = 52/238) were found. For this species, adults were found both in the intra and in the peridomicile, and nymphs only in the last. However, only adults were infected in both areas. The data related to this species indicate the non-formation of colonies in the intradomicile and, although existing, the risk of vector transmission can be considered low, since its infection rate by *T. cruzi* was 7.6% (n = 18/238). However, this rate almost doubled in relation to the previously investigated period, in which *P. geniculatus* had an infection rate of 4.7%.^23^ These data suggest that this species deserves vigilance so that it does not adapt to artificial ecotopes, developing the capacity to form colonies in the intradomicile, as occurs with *P. megistus* in Paraná, leading to an increase in its presence in the intradomicile and in the rate of infection by *T. cruzi*, since it has great potential to adapt to different ecological conditions.^48^

*Triatoma sordida* was the third most common species found in the current study, corresponding to 3.3% (n = 58/1,750) of the captured specimens. The majority, 60.3% (n = 35/58), were found in the intradomicile with a *T. cruzi* infection rate of 1.7% (n = 1/58), only one positive adult intradomicile. Over the period of this study, the largest number of captured specimens of this species was in 2012, ceasing to be found in subsequent years and reappearing in recent years. In the period of the previous study, it ranked 4th in Paraná, with most specimens also captured in the intradomicile and with the same infection rate.^23^ However, this species of triatomine was already the most often found in the state in the 90’s, usually with high rates of infection by *T. cruzi* in the peridomicile.^21^ ^,^ ^22^ Despite being associated with bird nests, which are refractory to infection by trypanosomatids, and small mammals,^55^ the *T. sordida* species is considered epidemiologically important in the transmission of CD to humans for invading houses and being positive for *T. cruzi*.^56^

Among the four most prevalent triatomine species, *R. neglectus* was the lowest occurrence in Paraná during the period studied, for which only adult specimens without *T. cruzi* infection were captured. These data suggest the absence of colonisation both in the intra and in the peridomicile and, so far, a low risk of *T. cruzi* transmission by them in the state. *Rhodnius* species are primarily associated with palm trees and the ENM approach has been used previously to generate suitability maps for *R. neglectus* and *T. sordida*, associating these species with the Cerrado biome, which in the State of Paraná occurs in only 1% of its area, which may be contributing to the low number of occurrences.^13^ ^,^ ^23^

In the environmental suitability maps for the occurrence of triatomine vectors generated by Ferro e Silva et al.^23^ using previously selected climate and landscape variables, a spatial prediction of areas at risk for vector transmission of *T. cruzi* was obtained using entomological data from SESA for the seven-year period, from 2007 to 2013. While in the present study, the same climate and landscape maps from Ferro e Silva et al.^23^ were used, but now with new triatomine capture data for the 10-year period, from 2012 to 2021. It was observed that the demarcated areas with the greatest climate and landscape suitability for the occurrence of triatomines in Paraná coincided with the areas with the highest occurrence of these insects, and the new data validate the general patterns estimated for the previous period, indicating high values for northern municipalities. The opposite was observed for areas with less environmental suitability that had a lower number of occurrences or zero occurrences. This demonstrates an adequate prediction for the risk areas of the maps generated by the previous study and current predictions.

The states of Paraná, Santa Catarina and Rio Grande do Sul are located in the Southern region of Brazil. During the 15-year period in which the two studies were carried out, between 2007 and 2021, although new acute cases of CD occurred in Rio Grande do Sul (three cases), no new cases were reported in Santa Catarina and Paraná.^4^ However, chronic cases of CD are frequently underreported, which further emphasises the disease’s neglected status. Moreover, numerous municipalities of the state remain at high zoonotic risk of vector transmission (see Table), despite slight variations in maximum values for suitability using new data for both climate and landscape models.

Municipalities in the northwest, north and northeast of the State of Paraná present higher risk of vector transmission of *T. cruzi*, which is consistent with regions that have high values of climatic and landscape suitability for the occurrence of vectors. On the other hand, the areas of the state that presented low values of environmental suitability values, South and East, also presented a lower concentration of municipalities with the occurrence of triatomines. Guamiranga was the municipality with the highest number of insects captured in both studies (n = 196 and 133 specimens, respectively, in the previous and current studies). Interestingly, the municipalities that had the highest amounts of captured insects, in addition to Guamiranga, Nova Aurora (n = 131), Boa Esperança (n = 106) and Prudentópolis (n = 91), were located in areas with high climate and high/medium landscape suitability (Table).

The literature points to the importance of temperature for the distribution of triatomines, indicating greater dispersion of vectors at higher temperatures.^13^ ^,^ ^57^ In the previous study, the proposed prediction models pointed out that the northwest, north and northeast areas were more suitable for the occurrence of triatomines, as they have such climatic characteristics.^23^ When analysing the mean suitability values for the climate ENMs, in the current study, it was found a low variation (0.70 to 0.84), indicating a good prediction and that the occurrences remained in more environmentally suitable areas.

The range of variation of average suitability values for landscape was greater (0.54 to 0.83) and several landscape factors can influence the occurrence of species, such as the type of vegetation, influencing the ecotope that the species may occupy, being it natural or artificial and the areas of connection between them, limiting its distribution. Areas of seasonal forest and mixed rainforest, predominant in Paraná, are highly suitable for the occurrence of triatomines.^23^ ^,^ ^30^

The natural habitat of some species of triatomines are nests of birds and mammals, in addition to occupying trees, such as palm trees.^58^ However, with several extractive actions due to deforestation, these insects were forced to migrate and occupy places close to human habitation, however, away from large urban centres. These insects were led to feed on domestic animals and humans, thus allocating themselves in areas inside and outside the home.^55^ The SAMPBIAS software pointed out locations close to highways as the most frequent areas for sampling triatomines. However, this bias may also be related to the methodology used to obtain latitude and longitude data (centroids) since these are not recorded by the health department and having this type of data would be of great importance in inferring the locations of species occurrence through spatially-explicit observations.

Entomological surveillance of triatomines can promptly identify characteristic situations of vector transmission, such as the detection of species capable of colonising the intra and peridomicile, in addition to monitoring the behaviour of these species over time.^10^ In Paraná, this surveillance occurs passively through the involvement and participation of the population,^25^ ^,^ ^59^ and this is why it is important to survey these records and disseminate them.

The results presented here suggest that the zoonotic risk for CD in Paraná remains high despite the absence of new reported cases in humans. Importantly, as presented by Rafael et al.^60^ the panorama of CD surveillance in an endemic region in southeast Brazil, it was possible to observe caveats regarding the program and surveillance and disease control activities. Since the decentralisation of the CD Control Program in 1990, passing responsibility for the program’s actions to the states and municipalities, there has likely been a loss of power in the program as actions are taken in a more isolated way, potentially including lack of prioritisation for the programs. Furthermore, in 2006, the control of the main vector of CD, the species *T. infestans*, was announced, establishing the likely false idea of eradication of the disease, and leading to a posterior lack of surveillance to it.

Therefore, the careful analysis of the relationship between the occurrence of triatomines and the presence of these insects found by the population is extremely important, which may have influenced our inferences of the areas with the highest occurrence. To get around this problem, information bulletins could be made available to the population of the State of Paraná encouraging them to forward the insects found to the nearest health department, intermediated by the Triatomine Information Points (PITs).

With zoonotic risk remaining high, it is paramount that surveillance and prevention strategies are kept in place in most of the state, especially in the municipalities where the models here described predict high climate and landscape suitability for the vectors. Moreover, educational strategies on how to avoid contact with the insects must be communicated year-long, including information and provision of better livelihood by, for instance, supporting adequate walls and making housing less precarious to the population at risk in the areas of higher zoonotic risk. The current results demonstrated that the ENMs were useful to predict the potential habitat and thus occurrence of triatomines in the State of Paraná, for both landscape and climate models. In this context, modelling may help to better understand the epidemiology of the disease and the geographic distribution of these vectors, as well as the *T. cruzi* parasite,^29^ ^,^ ^31^ ^,^ ^61^ demonstrating possible correlations to determine actions taken and guide the disease surveillance and control strategies. Furthermore, Ribeiro-Jr et al.^62^ cite the importance of analysing entomological data for vector control and surveillance. However, it is important to emphasise that bias analyses must be taken into account, considering that sample records presented in this study are in places with greater access (highways), and this likely is due, in part, to the use of municipality centroids, which ends up biasing the analysis and limiting the study to a restricted scale view of dispersal potential, especially for larger municipalities. Still, based on data aggregated to municipality through zonal statistics, the creation of maps, such as the ones modelled here, can be useful for investigating the large-scale dynamics of *T. cruzi* vector distribution and zoonotic risk in Paraná. Still, the existence of this bias reinforces the importance of recording the geographic coordinates of collection sites and supports the creation of an information system for this purpose, which would enrich and provide a more refined analysis of the niche dimensions and dispersal potential of each species found and its interplay with the population at risk.
