# Supplementary material for: The risk of vector transmission of Trypanosoma cruzi remains high in the State of Paraná
Source: Mem Inst Oswaldo Cruz. 2024 Jun 10;119:e230226. doi: 10.1590/0074-02760230226 (PMC11164317; doi:10.1590/0074-02760230226)
Supplement: Supplementary file 1 [file 1678-8060-mioc-119-e230226-s.pdf]

TABLE

Differences between data from Ferro e Silva et al.[23] and Trovo et al. (present study)

Year of study	2007 - 2013 (7 years)	2012 - 2021 (10 years)
Total number of specimens captured	2,662	1,750
Total, percentage (%) and number of specimens by species	Five<br>*Panstrongylus megistus* 73 (n = 1,943)<br>*Panstrongylus geniculatus* 15.4 (n = 411)<br>*Rhodnius neglectus* 6 (n = 159)<br>*Triatoma sordida* 4.5 (n = 119)<br>*Rhodnius prolixus** 1.1 (n = 30)	Six<br>*Panstrongylus megistus* 80 (n = 1,399)<br>*Panstrongylus geniculatus* 14 (n = 238)<br>*Triatoma sordida* 3 (n = 58)<br>*Rhodnius neglectus* 2 (n = 39)<br>*Rhodnius cis-prolixus* 1 (n = 14)<br>*Panstrongylus tibiamaculatus* < 1 (n = 2)
Infection rate (%, n) by *Trypanosoma cruzi*	19.7 (486)	22.7 (397)
Intradomicile (%, n)	71.9 (1,915)	57.7 (1,010)
Peridomicile (%, n)	26.9 (717)	42.3 (740)
Infection rate (%, n) in the intradomicile	NO	22.3 (225)
Infection rate (%, n) in the peridomicile	NO	23.3 (173)
Adults (%, n)	NO	73.7 (1,272)
Nymphs (%, n)	NO	27.4 (478)
Infection rate (%, n) of adults in the intradomicile	NO	21.4 (196)
Infection rate (%, n) of adults in the peridomicile	NO	16.3 (58)
Infection rate (%, n) of nymphs in the intradomicile	NO	30.9 (29)
Infection rate (%, n) of nymphs in the peridomicile	NO	29,9 (115)
Dispersion	*Panstrongylus megistus*<br>wide<br>*Panstrongylus geniculatus*<br>more restricted<br>*Triatoma sordida*<br>*Rhodnius neglectus*<br>*Rhodnius prolixus**<br>northwest and central-east	*Panstrongylus megistus*<br>wide<br>*Panstrongylus geniculatus*<br>more restricted<br>*Triatoma sordida*<br>*Rhodnius neglectus*<br>northwest and central-east
Main similarities and differences	**Similarities**<br>Some municipalities in the northwest, north and northeast of the Paraná state have higher risk of *Trypanosoma cruzi* vector transmission	**Similarities**<br>Some municipalities in the northwest, north and northeast of the Paraná state have higher risk of *Trypanosoma cruzi* vector transmission.<br>**Differences**<br>Identification error discussed of *Rhodnius prolixus*<br>New record identification<br>*Panstrongylus tibiamaculatus*<br>Climate and landscape ENMs: when overlapping the climatic and landscape layers for the different species found, we observed the current results show that, in recent years, a reduction in the maximum values for potential habitat suitability.<br>Sampling bias analysis: the triatomine occurrence data in the State of Paraná are biased towards the most accessible locations (close to highways) since the posterior weight of this variable is considerably higher than what would be expected for a random spatial sampling.

*Probable misidentification; NO: not observed.

CC BY
